# Editorial: Transcriptional regulation of glucose metabolism: gaps and controversies, volume II

**DOI:** 10.3389/fendo.2024.1383690

**Published:** 2024-02-27

**Authors:** Daniela P. Foti, Antonio Brunetti

**Affiliations:** ^1^ Department of Experimental and Clinical Medicine, University “Magna Græcia” of Catanzaro, Catanzaro, Italy; ^2^ Department of Health Sciences, University “Magna Græcia” of Catanzaro, Catanzaro, Italy

**Keywords:** glucose homeostasis, gene transcription, insulin resistance, insulin signaling, transcription factors

In mammals, glucose homeostasis is tightly regulated to sustain energy supply to vital organs and tissues, ensuring individual health. Genetic and/or environmental disruption of glucose metabolism and homeostasis can lead to cellular dysfunction, contributing to endocrine and metabolic disorders, such as insulin resistance, diabetes mellitus, the metabolic syndrome, and obesity (1–3). External signals, including hormones, adipokines, nutrients, and other biologically active molecules can influence cellular glucose uptake and utilization by modulating key enzymes directly involved in glucose-related metabolic pathways (4–6). Moreover, both ubiquitous and tissue-specific transcription factors, like ATF3, HMGA1, PPARγ, ChREBP, FOXO1, and PDX-1 are involved in several physiological processes and play essential roles in glucose metabolism and homeostasis (7–10). Furthermore, accumulating lines of evidence emphasize the important regulatory roles of non-coding RNAs, like miR-33, miR-34, miR-194, the newly discovered miR-128, and lncRNA-H19, which have been reported to impact glucose metabolism (11–13). However, the roles of other non-coding RNAs identified to date, remain largely unknown within this scenario (14, 15).

This second volume of “*Transcriptional regulation of glucose metabolism: gaps and controversies*” aims to expand understanding in this field.

Phosphatidylinositol 3-kinases (PI3K) represent a group of phylogenetically conserved enzymes that function as intracellular signal transducers, which are involved in many fundamental biological processes, including insulin signal transduction, cell growth, cell proliferation and differentiation (16). In mammals, the diverse range of isoforms found for each PI3K subunit (and their combinations) have likely evolved to adequately face the complexity of nutrient sensing and metabolic regulation in multicellular organisms. In this regard, Kim et al. provide an overview of the distinct roles of the regulatory subunits of class IA PI3K, and in particular the p85 regulatory subunit, in metabolism, cancer and immunity. Despite the structural similarities between p85α and p85β, the authors highlight their distinct functional repertoires in consideration of cellular stoichiometry (the relative abundance of p85 compared to the PI3K catalytic subunit, p110), tissue types, and external elements, like nutrients and insulin, concluding that future research is needed to better understand their physiological roles and their potential as drug targets.

The upcoming contributions to this topic share the interest in discussing or investigating some pathogenetic mechanisms underpinning specific metabolic diseases.

As the liver plays a key role in glucose and lipid metabolism, non-alcoholic fatty liver disease (NAFLD) has a bidirectional association with components of the metabolic syndrome, thus representing a risk factor for many metabolic diseases, including type 2 diabetes (T2D) (17). In their review article, Yu et al. discuss the role of members of the forkhead box (FOX) transcription factors in the context of hepatic glucose and lipid metabolism and in NAFLD. Being FOXA1, FOXA2, and FOXA3 involved in multiple stages of mammalian life (18), the authors offer a description of their role in the development of endoderm-derived organs, such as liver, pancreas and adipose tissue, in the control of glucagon and glucagon-like peptide-1 gene promoters, as well as in hepatic glucose and lipid metabolism. Contrary to what has emerged from studies in animal and cellular models, however, the precise role of FOXA proteins in NAFLD remains elusive.

Maturity Onset Diabetes of the Young (MODY) is the most common type of monogenic diabetes, affecting 1-5% of all diabetes cases (19). With the application of genetic sequencing techniques to clinical practice, and the greater awareness of this clinical entity among clinicians, especially endocrinologists, new candidate genes have been recently identified, further expanding our knowledge about this disease. In their review, Samadli et al. describe both traditional and emerging genetic determinants leading to MODY. They specifically categorize discrete etiological subtypes, ranging from defects in glucose sensing to channelopathies, impaired insulin trafficking, defects in transcriptional regulation, and even defects beyond the β -cell, affecting the interplay between pancreatic acinar and islet cells through mechanisms of exocytosis and endocytosis. Progress in this field holds promise to contribute, from a single protein perspective, not only to a better understanding and a tailored treatment of each MODY subtype, but also to sorting out the more complex mechanisms that characterize the common polygenic forms of diabetes.

Pancreatic islet cell failure is central to T2D development and progression. Song et al. utilize “omic” techniques and bioinformatics to explore potential crucial genes and pathways associated with pyroptosis and immune infiltration in islet dysfunction (20, 21). By combining a conjoint analysis of three bulk RNA-seq datasets, and single-cell transcriptome analysis of islet tissue, the authors identify immune and T2D-related differentially expressed genes associated with pyroptosis and immune infiltration, that may contribute to islet inflammation during T2D development. The discovery of distinct gene signatures through “omic” techniques can help unraveling new pathogenetic mechanisms, and may offer an important contribution to current knowledge of islet dysfunction and to future targeted therapeutic interventions in diabetes.

In summary, this Research Topic emphasizes the importance of elucidating the regulatory mechanisms operating in glucose metabolism and homeostasis. By introducing new players and utilizing “omic” techniques, the studies in this topic provide valuable insights into the regulation of genes, enzymes, and intracellular biomolecules involved in glucose metabolic pathways and energy storage. They also may help to identify potential therapeutic targets for improving glycemic control.

## Author contributions

DF: Writing – original draft, Writing – review & editing. AB: Writing – original draft, Writing – review & editing.
